# Respiratory Health Effects of Pollution Due to Artisanal Crude-Oil Refining in Bayelsa, Nigeria

**DOI:** 10.5334/aogh.4247

**Published:** 2023-10-27

**Authors:** Benson Chukwunweike Ephraim-Emmanuel, Okokon Enembe, Best Ordinioha

**Affiliations:** 1 Africa Centre of Excellence for Public Health and Toxicological Research (ACE-PUTOR), University of Port Harcourt, Rivers State, Nigeria; 2 Department of Community Medicine, University of Calabar, Calabar, Nigeria; 3 Department of Environmental Health, School of Public Health, University of Port Harcourt, Rivers State, Nigeria

**Keywords:** Crude oil, artisanal refining, environmental exposures, respiratory health symptoms

## Abstract

**Purpose:**

Artisanal refining of crude oil has been associated with the manifestations of various health problems directly related to the release of particulate matter, including polycyclic aromatic hydrocarbons (PAHs), into the environment. This study thus assessed the respiratory health effects associated with being resident in areas where crude oil is artisanally refined in Bayelsa State.

**Material and methods:**

This study utilized a comparative, cross-sectional design and was conducted in three communities in Bayelsa State. These included Sampou (a mildly exposed community), Nembe, and Gbarain (severely exposed communities). A sample population of 615 adults selected by multistage sampling completed the study instrument, which assessed data on their respiratory health. Environmental monitoring of the PAHs levels of the samples was done, and concentrations were determined using the gas chromatography/flame ionization detector (GC/FID). The Statistical Package for Social Sciences version 25 was used to conduct descriptive and inferential analyses.

**Results:**

Findings revealed that the highest number of moderate to severe respiratory disease symptoms was experienced by respondents from Nembe 12 (41.4%), followed by those from Sampou 8 (27.6%), and then by those from Gbarain 9 (31.0%). Also, coughing that occurred mostly when lying down was found to be significantly prevalent among residents of Nembe [35 (47.9%); p-value: 0.016], among other symptoms. Respiratory disease symptoms were more likely to be found among females (p-value: 0.037), smokers (p-value: 0.002), and those having a low health risk perception related to PAHs exposure (p-value: 0.002).

**Conclusion:**

Respondents from the three study sites had in the past 12 months experienced various respiratory disease symptoms, which could be directly related to their exposure to pollution from artisanal crude oil refining. Artisanal refining of crude oil should be continually dissuaded through unwavering enforcement of environmental health laws in order to further improve public and environmental health.

## Introduction

Crude oil exploration and exploitation in Nigeria have gone on for years and largely contributed to the Nigerian economy. This, however, occurs at the expense of the depletion of the environment and the occurrence of health problems, which are traceable to a number of events. These include the occurrence of accidental oil spills during oil exploration and transportation activities, gas flaring, fire outbreaks at exploration sites, as well as illegal oil bunkering activities. [1,2]. Illegal oil bunkering has been described as a very common form of crude oil theft, which usually involves sabotage of oil pipelines and the diversion of crude oil from these pipelines. This crude could be transported in barges to large offshore tanks, or it could be refined in make-shift refining facilities [2,3]. This unlicensed and illicit refining process is known as artisanal refining of crude oil. It is a highly inefficient means of refining crude oil as a result of its inability to refine as much as 80% of the heavy end of the crude and its tendency to produce refined petroleum products of low quality. This heavy end of the crude is discharged into the environment, where it causes an inadvertent destruction of the ecosystem and poses a serious health threat to human life as a result of combustible volatile compounds released into the atmosphere [4].

Artisanal refining involves the boiling of crude oil and the collection and condensation of resultant fumes in tanks to be used locally for lighting, energy sources, or transport [5]. The distilleries are heated on open fires, which are fuelled by crude oil that is tipped into pits in the ground. As part of the oil burns away, some seeps into the ground, with the potential to contaminate the underground aquifer [6]. During the course of the refining, dense clouds of soot, gaseous, and particulate compounds are produced and released together with the unrefined portions into the environment. Carbon black and soot are potential air pollutants produced in large amounts as a result of artisanal refining activity, and a major constituent of these pollutants is polycyclic aromatic hydrocarbons (PAHs) [6,7,8,9]. Environmental and health risks can emerge from the contamination of the environment with these pollutants. These contaminants have been associated with human carcinogenicity and tissue toxicity, and the unregulated release of these compounds into the environment poses a serious health and development risk to local populations [4,10,11].

A number of health abnormalities have been associated with the release of these kinds of volatile organic compounds into the environment when oil pollution occurs. These include cardiovascular, gastrointestinal, neurological, respiratory, psychological, visual, hematological, and carcinogenic pathologies [12,13]. Health problems related to artisanal refining directly affect the artisanal refiners themselves due to their immediate exposure to toxic products during the course of their operations [6]. These problems inadvertently spread to the surrounding populations as the toxic by-products of the refining process seep into the environment. Studies have identified these problems to include lung cancers [14], mutation of lymphoblast cell lines of the blood [15], respiratory diseases, including chronic obstructive pulmonary diseases, and cardiovascular diseases [8]. Some studies have reported respiratory function impairment and ocular irritations due to the burning of biomass fuels [16], and yet others have associated oil pollution with respiratory, cardiovascular, psychological, and pharyngeal irritation problems [6,12,13]. Only scant literature addresses the contribution of PAHs, which are released from artisanal refining activities, to human health problems in Nigeria.

There is, thus, a limited grasp of the human health burden of these activities in oil-producing communities. Considering the high incidence of crude oil theft and artisanal refining reported within the Niger Delta region of Nigeria [1,2,6,17], it is possible that the problems associated with illegal refining activities exist in Bayelsa State, being one of the states located in this region [2]. In the state, settlements are located along freshwater creeks, which the populace drinks from and carries out other activities with, given the low salinity levels. This inadvertently increases their interaction with the water bodies and increases their exposure to the pollutants in them [18]. This study thus set out to determine and compare the disproportionate respiratory health impacts of exposure to pollutants from artisanal refining activities, among residents of oil-producing communities in Bayelsa State, Nigeria. It also determined the association between PAHs pollutants concentrations in environmental samples and the presence of these respiratory symptoms.

## Materials and methods

This was a cross-sectional comparative study that was carried out in communities that have been impacted by artisanal refining of crude oil and gas flaring activities, as well as in a community where neither of these activities have been done. Sites included Ogbolomabiri in Nembe LGA, which has been impacted by artisanal refining activities, and Gbarain in Yenagoa LGA, which has been impacted by both artisanal refining and gas flaring activities and was regarded as being severely exposed to the PAHs pollutants in this study. The third study site was Sampou in Kolokuma/Opokuma LGA, which has neither been impacted by artisanal refining nor gas flaring activities and was regarded as a community mildly exposed to PAHs pollutants. This reference community was regarded as a mildly exposed site considering its downstream geographical relationship with communities in the bordering Delta and Rivers states where crude oil exploratory activities are carried out as well [4].

### Study population

The study population comprised healthy persons, both male and female, who had resided in the communities for not less than a period of 2 years. This time period of residence was chosen as it provided a good picture of chronic exposure to PAHs pollutants within the study areas. They were also aged between 18 and 65. Young and middle-aged adults were included because they represented an ideal population that has been sufficiently exposed to PAHs and in whom the physiological decline of lung functions has not set in. Individuals with a history of respiratory pathologic conditions or who had experienced major lung surgeries or thoracic trauma that affected lung function were excluded from the study, as were all individuals who were affected by a severe illness at the time of the study. Also, occupationally exposed individuals were excluded.

### Sample size determination

Formula for sample size for comparing two proportions was used in calculating the sample size used for this study [19].

The formula is shown below:

\[n = \frac{{{{(V + U)}^2} \times [P1\left({100 - P1} \right)] + [P2\left({100 - P2} \right)]}}{{{{[P1 - P2]}^2}}}\]

Where:

n = minimum sample size required for each group, V = standard normal deviation at a confidence level of 95% which is 1.96; U = standard normal deviation for statistical power at 80%, which is 0.84; P1 = proportion of the attribute of interest (respiratory symptoms) in a PAHs exposed group = 28.0% [20]; P2 = Proportion of the attribute of interest (respiratory symptoms) in a PAHs non-exposed group = 17.0% [20]. This yielded a total of 205, which was multiplied by three to give 615 (accounting for the comparison of three groups). The sample size was equally distributed across the three selected communities. Multistage sampling was used in the selection of the study respondents from the different communities. The first stage was simple random sampling to randomly select wards in the Local Government Areas (LGAs) by balloting. The second stage was simple random sampling by balloting to select communities within each selected ward. The third stage involved simple random sampling to select houses within selected communities. Each house formed a cluster, and everyone who met the inclusion criteria was recruited. Where more than one household was present in a house, simple random sampling by balloting was used to select only one household. While sampling, when any respondent chose not to participate in the study, sampling was extended to compensate for that. Sampling continued until the minimum sample size was met.

### Study Instrument

A detailed self- and interviewer-administered questionnaire was used to elicit the respondent’s data. The questionnaire was adapted from existing questionnaire templates from previous studies [13,20,21]. The questionnaire was divided into four different sections (A to D). Section A elicited data on the demographics of the respondents. Section B elicited individual-level covariates, such as lifestyle factors, which could act as confounding factors in this study. Section C elicited data on the respondent’s respiratory health, and section D elicited data on their perception of the health and environmental risks associated with the artisanal refining of crude oil.

### Outcome assessment

The outcome in this study was the chronic respiratory symptoms of the study participants, which were assessed by asking a series of questions designed to elicit symptoms of respiratory disease in a survey scenario [22,23]. Scores from the symptoms reported by the subjects were added to a composite score for each study participant. A score of between 1 and 5 was regarded as having mild (respiratory) ill-health; a score of between 6 and 10 was regarded as having moderate (respiratory) ill-health; and a score of between 11 and 15 was regarded as having severe (respiratory) ill-health.

### Data analysis

The Statistical Package for Social Sciences (SPSS) version 25 (IBM, Armonk, New York, USA) was used to perform both descriptive and inferential analyses using the collected data. Preliminary exploration of the data was done to identify outliers, check the normality of continuous variables using the Kolmogorov-Smirnov normality test, identify any patterns of missingness in the data, and assess the appropriateness of transforming variables. Missing entries were assessed by comparing the socio-demographic variables of those who provided the entry with those who did not to ascertain if there was systematic missingness.

The Chi-squared test was used to compare the occurrence of respiratory symptoms among the three LGAs. The multivariable logistic regression was then used to assess the relationships between PAHs pollutants’ exposures (relative to the study communities) and the dichotomous outcomes of the presence and absence of respiratory toxicity symptoms. The results from the fully adjusted models were presented. The fully adjusted models were adjusted for age, sex, educational attainment, smoking, and the perception of health risk. All analyses were conducted at the 95% confidence level, and a p-value ≤ 0.05 was considered statistically significant.

### Ethical considerations

Ethics approval for the research was obtained from the Research Ethics Committee of the University of Port Harcourt (approval number: UPH/CEREMAD/REC/MM72/097). Permission to conduct the research was also obtained from the Bayelsa State Ministry of Health (approval number: BSHREC/Vol. 1/21/02). Permission to conduct this study was also sought from the necessary authorities of the communities involved. Every part of the research protocol was explained to the respondents, and their consent was sought and obtained before the commencement of instrument administration. The decision to partake in the research was completely voluntary. Respondents’ confidentiality, necessary to ensure honest answers from participants, was ensured. They were also assured that all information being provided by them was strictly used for the purpose of research and treated as confidential.

## Results

### Socio-demographic characteristics of respondents

Out of the 615 respondents who took part in this study, 205 were selected from each community. It was found that the majority of the respondents were male, 318 (51.7%), and the majority of the respondents were aged between 18 and 44 years, 490 (82.9%), with a mean age of 32.77 ± 11.13 years. The largest proportion of the respondents were single, 281 (45.7%) and self-employed, 290 (47.2%). Most of the respondents had received secondary school education, 319 (52.3%). These details are shown in Table 1.

**Table 1:** Demographic data of respondents.


VARIABLES	FREQUENCY	PERCENTAGE (%)

Sex		

Male	318	51.7

Female	297	48.3

Total	615	100

Age (years)		

18–44	490	82.9

45–60	90	15.2

>60	11	1.9

Total	591	100

Marital status		

Single	281	45.7

Married	270	43.9

Divorced	17	2.8

Widow(er)	8	1.3

Cohabiting	39	6.3

Total	615	100

Employment status		

Unemployed	208	33.9

Self-employed	290	47.2

Employed by others	116	18.9

Total	614	100

Educational qualification		

None	30	4.9

Primary	27	4.4

Secondary	319	52.3

Tertiary	206	33.8

Vocational/Technical	28	4.6

Total	610	100


### Prevalence of respiratory disease symptoms among the respondents

**Figure 1:**
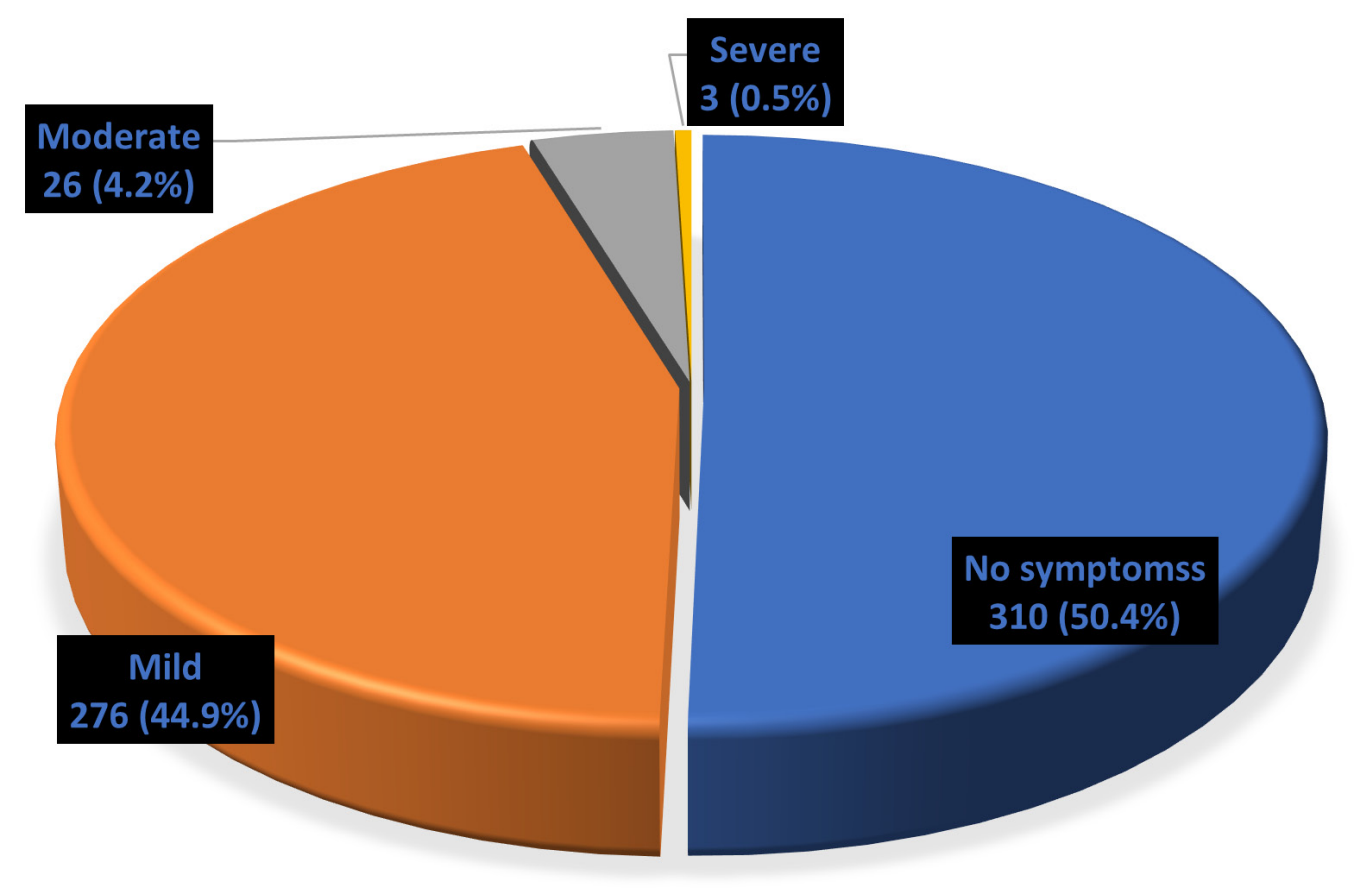
Resp disease symptoms.

Assessment of the prevalence of respiratory disease symptoms among the respondents in the past 12 months showed that up to 305 (49.6%) of the respondents cut across the three communities had experienced these symptoms. 276 (44.9%) of the respondents had experienced mild respiratory disease symptoms, 26 (4.2%) had experienced moderate respiratory disease symptoms, and 3 (0.5%) had experienced severe respiratory disease symptoms. This is as shown in Figure 1.

### Prevalence of respiratory disease symptoms according to communities

When respiratory symptoms were stratified by the communities of the respondents, it was found that the majority of the respondents from Sampou, 102 (37.0%), reported mild respiratory disease symptoms within the past year, compared to 95 (34.4%) from Nembe and 79 (28.6%) from Gbarain. Furthermore, the highest number of moderate to severe respiratory disease symptoms was experienced by respondents from Nembe 12 (41.4%), followed by those from Sampou 8 (27.6%), and then by those from Gbarain 9 (31.0%). This distribution is shown in Table 2.

**Table 2:** Prevalence of respiratory disease symptoms according to communities.


VARIABLES	SAMPOU	NEMBE	GBARAIN	TOTAL	X^2^ (p-VALUE)

SYMPTOMS OF RESPIRATORY DISEASE	FREQ (%)	FREQ (%)	FREQ (%)	FREQ (%)	df

No symptoms	95 (30.6)	98 (31.6)	117 (37.7)	310 (50.4)	6.673 (0.154)

Mild symptoms	102 (37.0)	95 (34.4)	79 (28.6)	276 (44.9)	df = 4

Moderate to severe symptoms	8 (27.6)	12 (41.4)	9 (31.0)	29 (4.7)	

**Total**	205 (33.3)	205 (33.3)	205 (33.3)	615 (100.0)	


X^2^: Chi-squared.

### Prevalence of specific respiratory disease symptoms among the respondents

Assessment of the prevalence of the specific respiratory disease symptoms experienced among the respondents in the past 12 months showed that 215 (35.0%) of the respondents had experienced chest pain when they breathed deeply, 148 (24.1%) had experienced coughing that produced thick sputum, and 73 (11.9%) had experienced coughing that occurred mostly when lying down. Furthermore, 9 (1.5%) had coughed up blood, 60 (9.8%) had coughs that woke them early in the morning, 36 (5.9%) had had trouble breathing, 48 (7.8%) had experienced breathlessness when walking fast, 20 (3.3%) had experienced breathlessness when walking normally, and 37 (6.0%) had experienced breathlessness when working. It was also found that 36 (5.9%) had a persistent stuffy nose, 51 (8.3%) had a persistent running nose, 37 (6.0%) had experienced thick mucus production without coughing, 17 (2.8%) had experienced whistling sounds from the chest, 37 (6.0%) had had chest tightness, and 17 (2.8%) of them had experienced episodes of struggling to breathe. It was also identified that a significant difference in the occurrence of respiratory symptoms among the communities severely exposed to the pollutants, occurred for coughing when lying down (p-value: 0.016), feeling breathless when performing ordinary activities like walking (0.02), and mucus build-up/production even without coughing (0.026). This is shown in Table 3.

**Table 3:** Prevalence of specific respiratory disease symptoms (multiple responses were given).


VARIABLES	SAMPOU	NEMBE	GBARAIN	TOTAL	X^2^ (p-VALUE)

	FREQ (%)	FREQ (%)	FREQ (%)	FREQ (%)	

Chest pain with deep breathing	75 (34.8)	73 (34.0)	67 (31.2)	215 (35.0)	0.744 (0.689)

Coughing that produces thick sputum	73 (49.3)	39 (26.4)	36 (24.3)	148 (24.1)	22.548 (<0.001)*

Coughing that occurs mostly when lying down	21 (28.8)	35 (47.9)	17 (23.3)	73 (11.9)	8.331 (0.016)*

Coughing up blood	0 (0.0)	5 (55.6)	4 (44.4)	9 (1.5)	4.736 (0.094)

Coughing that wakes early in the morning	23 (38.3)	25 (41.7)	12 (20.0)	60 (9.8)	5.430 (0.066)

Difficulty breathing	15 (41.7)	13 (36.1)	8 (22.2	36 (5.9)	2.301 (0.316)

Breathlessness when walking fast	14 (29.2)	20 (41.7)	14 (29.2)	48 (7.8)	1.627 (0.443)

Breathlessness when walking normally	2 (10.0)	6 (30.0)	12 (60.0)	20 (3.3)	7.855 (0.02)*

Breathlessness when working	15 (40.5)	9 (24.3)	13 (35.1)	37 (6.0)	1.610 (0.447)

Persistent stuffy nose	12 (33.3)	16 (44.4)	8 (22.2)	36 (5.9)	2.832 (0.243)

Persistent running nose	28 (54.9)	18 (35.3)	5 (9.8)	51 (8.3)	17.062 (<0.001)*

Thick mucus production without coughing	6 (16.2)	19 (51.4)	12 (32.4)	37 (6.0)	7.304 (0.026)*

Whistling sounds from chest	1 (5.8)	8 (47.1)	8 (47.1)	17 (2.8)	5.929 (0.05)*

Chest tightness	8 (21.6)	16 (43.2)	13 (35.1)	37 (6.0)	2.818 (0.244)

Episodes of struggling to breathe	5 (29.4)	6 (35.3)	6 (35.3)	17 (2.8)	0.121 (0.941)


Testing the association between the PAHs concentrations of the environmental samples and the presence of respiratory toxicity symptoms relative to the study communities showed that sex, perception of health risk score, and smoking were statistically significant predictors of the presence of these symptoms. Respiratory symptoms were more likely to be found among females (p-value: 0.037), smokers (p-value: 0.002), and those with a low perception of health risk due to PAHs exposure (p-value: 0.002). Also, though not statistically significant, Nembe (one of the severely exposed communities) presented with a higher risk of coming down with respiratory toxicity symptoms when compared with Sampou. This is shown in Table 4.

**Table 4:** Logistic regression of the prevalence of respiratory symptoms relative to the study community.


VARIABLE	CATEGORIES	p-VALUE	ODDS RATIOS	95% CONFIDENCE INTERVAL	

				LOWER	UPPER

Community	Sampou		1		

	Gbarain	0.261	0.768	0.485	1.216

	Nembe	0.596	1.127	0.725	1.751

Age		0.839	0.998	0.981	1.016

Sex	Females		1		

	Males	0.037*	**0.679**	**0.472**	**0.976**

Employment status	Unemployed		1		

	Self employed	0.665	1.103	0.708	1.718

	Working for another	0.362	1.335	0.717	2.487

Educational status	None		1		

	Primary	0.078	3.345	0.874	12.807

	Secondary	0.348	0.657	0.273	1.580

	Tertiary	0.275	0.598	0.237	1.505

	Vocational/Technical	0.736	0.815	0.248	2.675

Alcohol consumption	None		1		

	Occasional	0.381	1.557	0.579	4.190

	Habitual	0.697	1.221	0.447	3.333

Perception of health risk score	High	0.002*	**0.935**	**0.895**	**0.976**

	Low		1		

	No smoking		1		

Smoking	Past smoker	0.189	1.623	0.788	3.343

	Present smoker	0.002*	**4.279**	**1.680**	**10.898**


## Discussion

In this study, it was found that almost half of the respondents across all three communities had in the past 12 months experienced various respiratory disease symptoms ranging from mild to severe. When respiratory symptoms were stratified by communities, it was found that the majority of respondents from Sampou reported more mild respiratory disease symptoms within the past year compared to respondents residing in Nembe and Gbarain communities, where more moderate and severe respiratory disease symptoms were reported. It was also identified that there was a significant difference in the occurrence of some of the respiratory symptoms among the severely exposed communities. These symptoms included coughing when lying down, feeling breathless when performing ordinary activities like walking, and mucus buildup or production even without coughing. It is important to note that these are symptoms that have been pinpointed by the literature to be suggestive of heart failure in human populations [24].

The relative differences in the respiratory disease symptoms experienced in this study can be related to a number of factors. These include the duration, intensity, and frequency of individual exposures to the PAHs compounds in the different communities, especially those regarded as being severely exposed to pollutants from crude oil exploratory activities [25,26,27,28,29]. It could also have been a result of individual nutritional deficiencies [30], as well as genetic and molecular susceptibilities to disease among the different study respondents [8,31,32].

These findings are similar to the reports made in studies on soot pollution impacts in communities located within the Niger Delta region of Nigeria, where, among other complaints, there were complaints of respiratory tract diseases within the population [33,34,35,36]. A strong association has also been reported between soot-containing air pollutants, including PAHs (which make up particulate matter), and the incidence of various respiratory health abnormalities, including cough, sinusitis, bronchitis, and so on [37,38]. The direct effects of crude oil exploratory activities that release atmospheric pollutants have also been reported to include the occurrence of respiratory function impairment among affected members of the population as well as lung damage [13,39]. This was also reported in the cross-sectional study that assessed the respiratory symptoms of residents living in close proximity to a crude oil first treatment plant located in Italy. The study showed that the prevalence of respiratory disease symptoms increased with increased proximity to the plant [40]. Direct contact of these pollutants with the respiratory tract could also initiate oxidative and/or necrotic damage to the lung epithelial cells. Systemic toxicity due to exposure to these compounds is related to toxicities that manifest beyond the site of exposure [8].

In this study, it was also found that respiratory disease symptoms were more likely to be found among females, smokers, and those having a low perception of health risk related to PAHs exposure. Residence in one of the communities where artisanal refining of crude oil took place presented a higher risk of respiratory symptoms when compared with the mildly exposed community (Sampou). These associations have also been reported in other studies, which have reported that the female gender [41], smoking [42,43,44], and residence in areas that promote exposure to PAHs compounds [45,46] were associated with an increased likelihood of manifesting respiratory symptoms of disease. The finding of poor perception of the associated health risk as a predictor of the occurrence of respiratory health abnormalities was, however, not corroborated by the findings of other authors, who were of the opinion that having good perceptions of the health risk contributed to the occurrence of disease symptoms [13]. This contrary finding could be a result of the combination of other factors such as stress, worry, and annoyance levels in the assessment of factors having a contributory effect on the occurrence of poor health outcomes [13].

### Study limitation

In the assessment of the prevalence of respiratory symptoms among the respondents, this study used a series of questions designed to elicit the symptoms of respiratory disease. This approach was self-reported and could have been subject to recall bias on the part of the respondents at the time of the study. However, it was impossible to use a spirometer and unavoidable to utilize the approach used in this study for ethical reasons. This was as a result of the occurrence of the COVID-19 pandemic (characterized by respiratory-droplet transmission) during the period just before data collection.

## Conclusion

This study thus concludes that mild to moderate respiratory symptoms were experienced by up to 49.6% of the respondents, and the highest proportion of respondents who experienced more moderate and severe respiratory disease symptoms were residents of one of the severely exposed communities (Nembe). Respiratory symptoms were predicted to be more likely found among females, smokers, and those with a low perception of health risks related to PAHs exposure.
